# The interplay between mental health and dosage for gaming disorder risk: a brief report

**DOI:** 10.1038/s41598-024-51568-9

**Published:** 2024-01-13

**Authors:** Paweł Strojny, Magdalena Żuber, Agnieszka Strojny

**Affiliations:** Institute of Applied Psychology, Faculty of Management and Social Communication, Jagiellonian University, Ul. Stanisława Łojasiewicza 4, 30-348 Kraków, Poland

**Keywords:** Human behaviour, Psychology, Health care

## Abstract

The relationship between gaming time and gaming disorder can be moderated by other variables. This study aimed to test the moderating role of mental health. Participants (N = 461) were recruited online. Gaming time was a statistically significant predictor of gaming disorder risk, with an explained variance of 3.3%. The goodness of fit of the model that took into account both moderators (anxiety and depression) improved to 13.9%. The interaction between gaming time and both moderators was significant. The results showed that depression and anxiety acted as moderators of the dosage effect, possibly by amplifying the gratification of playing games and thus contributing to the development of gaming disorder. It may be important in practise, as it seems to place the mental health at the right place, namely among risk factors that can contribute to gaming disorder in combination with a key trigger, which is gaming.

## Introduction

The vast majority of computer game users have not and will not experience gaming disorder^1^. The prevalence of gaming disorder (GD) varies^2^, but it can be assumed that in general populations it is approximately 3%^3^. The association between mere gaming time and GD can be called the dosage effect^4,5^. Although gaming time may be considered one of the most important variables with regard to gaming disorder, correlation coefficients vary between 0.17 and 0.4^1,6–9^. Empirical studies confirm the occurrence of the dosage effect, but the relationship is as consistent as it is only low, moderate at most^1^ and can depend on additional factors^10^. Therefore, researchers often look for additional factors that may explain some of the variance. They relatively often focus on mediators, but only in a few cases they look at moderation dependencies^11^. Moderators should be understood as variables that explain under what conditions two variables (e.g., gaming time and gaming disorder) are related^12^. Therefore, their identification leads to the identification of the real risk factors for GD. This article aims to test hypotheses on the moderating role of two variables often treated as related to GD, namely depression and anxiety.

Generalized depression and generalized anxiety are related to GD. Some researchers treat them as comorbidities^13^; others argue their more direct relationship with GD, most often as predictors^14^ or consequences^15^. Results suggesting the opposite direction of the relationship between GD and mental health also exist, for example, anxiety and depression, among others, were shown to be outcomes of gaming disorder^10^. This may suggest that mental health should be treated as a predictor, consequence, or mediator in GD models. This trend is also reflected in review articles listing the most common comorbidities of GD (including depression and anxiety). Their results are not clear; for example, González-Bueso^16^ reports that out of 15 studies with the measurement of depression, only in eight cases can the strength of the association be described as "large" (the criterion is a minimum of 14% of the explained variance or Cohen's d > 0.8), and in two cases no relationship has been found. Even less spectacular results were obtained from the analysis of the relationship between anxiety and GD. In this case, only two out of ten studies showed "large" effect sizes and one showed null effect. Taking into account the risk of publication bias identified by the authors, it is even more difficult to consider this direction of research conclusive. These results are corroborated by more recent studies; including a meta-analysis indicating that the correlations between GD and these two health disorders had an average small to medium effect size^17^ and a systematic review showing that depression symptoms are present in 32% of people at risk of GD^18^. It seems that the relationship between gaming disorder and anxiety (depression) is unclear, which may be due to various factors^19^. However, one of the reasons may also be the wrong direction of search. Therefore, the question should be asked whether the role of these two variables may be different. It is impossible to develop GD without using video games just because a person has a mood disorder. Perhaps an approach that allows for both a moderating and a mediating role of depression and anxiety in the development of GD may be useful. A similar dual moderation-mediation role is presented in the interactional-transformational model^20^ regarding the role of outcome expectancies in the development of substance abuse.

At this point, the question arises: Can the relationship between gaming time, GD, and depression or anxiety be of an interactional nature? Can anxiety or depression moderate (facilitate) the development of GD? The Person-Affect-Cognition-Execution Interaction (I-PACE) model^21^ postulates that the interaction between dysfunctional personality traits, psychopathological characteristics (eg, depression, social anxiety), other general factors (eg, vulnerability to stress) and behavior-specific factors (eg, ‘a strong predilection towards gaming”; p. 254) influences addictive behaviors by influencing the gratification resulting from specific activities^22^. Assuming, for simplicity, that all remaining factors are constant, according to the I-PACE model, psychopathology-related variables (such as anxiety and depression) can increase the level of gratification from gaming^21^, which in turn results in an increased risk of GD^23^. It follows that conditions related to anxiety and depression can promote deficits in behavioral control and thus contribute to increases in gaming. This may be due, for example, to focusing attention on short-term rewards and risky decision-making^24^. In other words, depression or anxiety can increase the level of gratification, a mediator directly responsible for the development of GD. Gratification may be related to treating gaming as a coping method, which may result from the interaction of general predisposing factors and behavior-specific predisposing factors (i.e., motives, needs). Therefore, they may be more likely to develop GD while maintaining similar gaming intensity to that of their healthy counterparts. It should be noted that the I-PACE model differentiates between the early and late stages of the development of addictive behavior development^20^; depending on the stage, the postulated role of anxiety and depression is different; At an early stage, the gratification mechanism described above occurs, and later mood disorders may result from compensatory mechanisms. This could explain the inconsistencies in the results obtained regarding the direction of the relationship between mood disorders and gaming disorder, which has been previously empirically demonstrated^25^. The first studies using the postulates of both models on GD showed that several factors moderate the relationship between gaming time and probability of GD. These factors were perceived urge and loneliness^4^, depression, ADHD, self-esteem, and gaming motivation^11^.

Based on this, we conducted a study aimed at verifying the moderating role of the two conditions most strongly associated with GD, namely depression and anxiety. We predicted that higher levels of both depression (hypothesis 1) and anxiety (hypothesis 2) would result in stronger associations between gaming time and GD among participants representing the general population of gamers, that is, participants who potentially may be at an early stage of development of a gaming addiction according to the I-PACE model and whose interests predispose them to gaming.

## Methods

### Participants

A survey was conducted among Polish gamers over 18 years of age who play video games on any device (computer, smartphone, console, tablet, or other). In total, 595 people participated. We assumed data elimination in two cases: when participants provided highly implausible information on their gaming habits (that is, more than 100 h per week or an average gaming session of more than 12 h) and in cases of completion of the study in less than 4 min. Fourteen responses were automatically excluded due to the criteria mentioned above and one response was eliminated due to homogeneous responses. Of the remaining 580 responses, 461 were complete and, therefore, could be used for analysis. The age range was 18–48; they completed the study (M_age_ = 23.5, SD_age_ = 5.1), including 200 (43%) women and 247 (54%) men; the rest of the respondents identified themselves as non-binary (8 people, 2%) or refused to answer the question (6 people, 1%).

### Procedure

Participation was entirely voluntary and no financial compensation was offered to participants. Data were collected from April to June 2022 using Qualtrics (www.qualtrics.com). Participants were eligible to participate in the consent study after answering the screening questions, being 18 years or older, and playing video games. Respondents were not allowed to take the survey if they responded ‘no’ to any of these questions. The recruitment was carried out using the snowball method and Facebook groups that associate players of various genres of games, with a few groups not themed with games. This method allowed for the differentiation of results and the recruitment of players from many different genres. The recruitment of groups not related to games was motivated by the desire to collect data not only from the players involved in the community, but also from those who do not spend time on activities related to games outside of the game itself.

### Measures

#### Background variables

Participants were asked about demographics such as gender, place of residence, age, level of education, and status of relationships.

#### Gaming behavior

Respondents answered questions about their gaming preferences. We asked about the dominant type of game in the last 12 months (online or offline), the genres of games they played in the last 12 months, and the preferred devices such as a computer or laptop, smartphone, console, and VR. There was also a self-diagnostic question about problem gaming (“I think I play games in a way that significantly impairs my functioning” with the answer options ‘yes’ and ‘no’). This question was intended to check the relationship between the perception of one's own problem and the occurrence of the real problem.

#### Gaming time

Respondents were asked about the average time they spend playing per week. The answer was given in hours. The average time spent gaming per week was 15.25 (SD = 12.2). The participants also answered the question of how long their average gaming session lasts in minutes. The results showed that the average gaming session lasts 143 min (SD = 92.32).

#### Gaming disorder risk

To measure the risk of gaming disorder among respondents, we use the Gaming Disorder Test^26^. A four-item Gaming Disorder Test was developed based on the diagnostic criteria for gaming disorder in the international classification of diseases ICD-11. The translation was done by one of the authors using the back-translation method by a translator unrelated to the work on the tool; the English version after back-translation has been approved by the author of the original. Cronbach’s alpha was α = 0.8. In our analysis, we used the sum of the answers for all items (varying between 4 and 20) as a continuous measure of the risk of GD. According to the authors of the tool, the occurrence of GD can also be determined binaryly in people who answered each of the four questions ‘Often’ (4) or ‘Very often’ (5).

#### Depression

We used the 9-item Patient Health Questionnaire (PHQ-9)^27^ in the Polish version^28^ to measure the level of depression. The questionnaire is intended to detect depression in the initial psychological diagnosis and is open-access. For ethical reasons, the last, ninth item, was excluded due to concern about the potential emotional trigger (question about self-harm and suicide). For this questionnaire, Cronbach’s alpha was α = 0.88.

#### Anxiety

Anxiety was assessed using the 7-item Generalized Anxiety Disorder Questionnaire^29^. The Polish version used in the study was created by MAPI Research Institute (www.phqscreeners.com). This scale is used to detect generalized anxiety disorder. Cronbach’s alpha for this scale was α = 0.89.

### Statistical analysis

The risk of gaming disorder was a dependent variable, and the gaming time was an independent variable. Since depression and anxiety tend to correlate strongly^30^, we decided to test the assumption of collinearity. Furthermore, following the recommendations of Gregorich et al.^31^ regarding the explanatory use of multiple regression, we decided to conduct regression analyzes in a model that takes into account both of these variables and in two separate models, treating each variable as an individual predictor/moderator. We verified the roles of both moderators separately and compared these models with the model that included both moderators. The difference in r^2^ of the two corresponding models (with and without interaction) was derived for each potential moderator. All statistical analyzes were performed with SPSS 28.0; To verify the moderation hypotheses, the PROCESS macro for SPSS^32^ was used. The statistically significant level was defined as two-sided *p* < 0.05. The information on moderation analysis will be separate from the information on hierarchical regression analysis in the Results.

### Ethical approval and informed consent

This study was performed in line with the principles of the Declaration of Helsinki. Approval was granted by the Research Ethics Committee at the Institute of Applied Psychology of the Jagiellonian University. Opinion number 102/2021. Informed consent was obtained from all individual participants included in the study.

## Results

### Descriptive statistics

The number of participants who met the binary GD criteria in our sample was 3 (0.52%). Data on gender, place of residence, age, level of education, and relationship status are summarized in Table 1.

**Table 1:** Demographic data of the respondents (N = 461). Note: the percentages for preferred genres do not add up to 100 because participants had the opportunity to mark all genres they played.

Categorical variables	n (%)
Gender	
Male	247 (53.6)
Female	200 (43.4)
Non-binary	8 (1.7)
I'd prefer to not answer	6 (1.3)
Place of residence	
Village	119 (25.8)
City up to 50,000 residents	66 (14.3)
City up to 100,000 residents	36 (7.8)
City up to 500,000 residents	240 (52.1)
Education level	
Primary	46 (10)
Vocational	17 (3.7)
Secondary	261 (56.6)
Higher	137 (29.7)
Relationship	
Yes	254 (55.1)
No	207 (44.9)
Preferred game mode	
Online	211 (45.8)
Offline	250 (54.2)
Genres used	
RPG	335 (72.7)
MMORPG	50 (10.8)
FPS	157 (34.1)
Simulators	149 (32.3)
Arcade	115 (24.9)
Strategy	143 (31.0)
Educational	15 (3.3)
Sport	38 (8.2)
Racing	43 (9.3)
MOBA	104 (22.6)
*Continuous variables*	Mean (SD)
Age (years)	23.53 (5.09)
Gaming per week (hours)	15.25 (12.21)
Length of single session (minutes)	142.73 (92.32)
GDT	7.42 (3.05)
Depression	6.81 (5.91)
Anxiety	5.47 (5.38)

The correlations between the variables analyzed can be found in Table 2.

**Table 2:** Correlations between analysed variables.

	1	2	3	4
Gaming per week	1			
GDT score	.168**	1		
Depression score	.082	.313**	1	
Anxiety score	.076	.252**	.824**	1

Pearson’s correlation coefficients are presented; N = 459 ** *p* < .01.

### Predictors of the risk of gaming disorder

A linear regression was performed to understand the effect of the average weekly time spent gaming on the risk of GD taking into account the control variables (age and gender). Linearity was assessed using partial regression plots and a plot of studentized residuals against predicted values. There was homoscedasticity, as assessed by visual inspection of a plot of studentized residuals versus unstandardized predicted values. There was no evidence of multicollinearity, as assessed by tolerance values greater than 0.1. There were 3 cases with studentized deleted residuals greater than ± 3 standard deviations, 3 others with leverage values greater than 0.2, and none with values for the Cook distance greater than 1. A careful inspection of the cases revealed that there were no errors that warranted case deletion, but analogous analyzes were performed after excluding these six cases to see if they influenced the results. The results obtained were very similar to those reported on the complete database. The assumption of normality was met, as assessed by a Q-Q plot. There was independence of residuals, as assessed by a Durbin-Watson statistic of 1.968. The dosage effect has been found; Game time turned out to be a statistically significant predictor of a risk of GD (F (3453) = 5.227, *p* < 0.001) and represented 3.3% of the explained variability in the risk of GD. Inclusion of depression and anxiety improved the fit of the model (*F*(5,453) = 12.275, *p* < 0.001. *r*^2^ = 0.119). However, after including both predictors in one model, only depression remained a statistically significant predictor of the risk of GD (see the details in Table 3).

**Table 3:** Hierarchical Multiple Regression Predicting the Risk of GD from Gaming Time, Depression, and Anxiety.

	Gaming disorder risk					
	Model 1		Model 2		Model 3	
Variable	B	β	B	β	B	β
Constant	7.80***		5.59***		6.08***	
Age	−.04	−.07	−.01	−.15	−.01	−.01
Gender	.04	.01	.29	.05	.28	.05
Gaming Time	.04***	.15	.03**	.13	−.00	.10
Depression			.16***	.31	.20***	.33
Anxiety			−.01	−.01	−.13	−.03
GT * Depression					−.00	−.06
GT * Anxiety					.01*	.17
*R*2	.033		.119		.14	
*F*	10.512***		12.275***		10.40***	
Δ*R*^2^	.03		.09		.02	
Δ*F*	10.512***		22.118***		5.16**	

N = 459 * *p* < .05, ** *p* < .01, *** *p* < .001.

#### Interaction of gaming time and psychopathology

In the case of all moderation analyzes described below, the assumption of homoscedasticity was met, as assessed by visual inspection of a plot of studentized residuals versus unstandardized predicted values. The tests to see whether the collinearity data met the assumption indicated that multicollinearity was not a concern (gaming time, tolerance = 0.99, *VIF* = 1.01; Depression, tolerance = 0.32, *VIF* = 3.13; Anxiety, tolerance = 0.32, *VIF* = 3.13) despite the high correlation of both variables (Pearson’s *r* = 0.82, *p* < 0.01). There was independence of residuals, as assessed by a Durbin-Watson statistic of 2.019. The model that took into account age and gender as control variables that took into account both moderators at the same time was statistically significant and its goodness of fit was further improved (*F*(7457) = 10.402, *p* < 0.001, *r*^2^ = 0.139). However, it turned out that only the interaction between gaming time and anxiety (but not depression) was statistically significant (see the details in Table 3, Model 3). Due to the fact that our study had explanatory rather than predictive objectives, in the following part of the text we decided to present in detail the results of both analyses taking into account depression and anxiety separately.

#### Interaction of gaming time and depression

**Figure 1:**
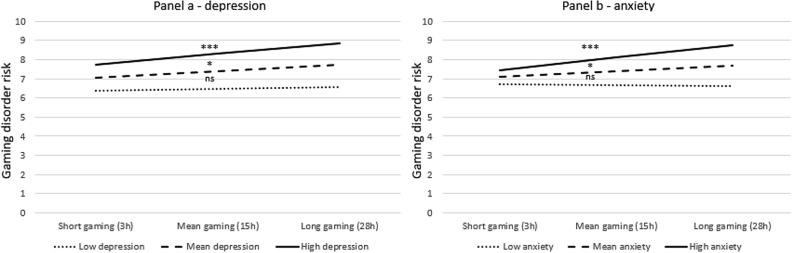
Gaming disorder risk as a function of the interaction between gaming time and psychopathology symptoms; depression (**a**) and anxiety (**b**). Note: *: *p* < .05; ***: *p* < 0.001; ns: non-significant.

According to Hypothesis 1, we analyzed the interaction between gaming time and depression symptoms (with control of age and gender). There was independence of residuals, as assessed by a Durbin-Watson statistic of 1.963. We found a statistically significant interaction (*b* = 0.0033, *SE* = 0.0016 *p* = 0.04, increase *r*^*2*^ = 0.008). Simple main effects indicated that in the case of depression at the mean level, the effect of the game time was significant (*b* = 0.027, *SE* = 0.012, *p* = 0.02, 95% CI: 0.004, 0.050). High depression resulted in a stronger dosage effect (*b* = 0.046, *SE* = 0.013, *p* = 0.0004, 95% CI: 0.021, 0.072). For low depression, the relationship was insignificant (*b* = 0.008, *SE* = 0.017, *p* = 0.66, 95% CI: −0.026, 0.041). This dependency is shown in Fig. 1, panel A. The Johnson-Neyman technique showed that a depression score of 6 and higher (calculated threshold: 5.87) resulted in a significant dosage effect. Two hundred and eleven participants scored above that threshold.

#### Interaction of gaming time and anxiety

According to Hypothesis 2, we analyze the interaction between gaming time and anxiety (with a control of age and gender). There was independence of residuals, as assessed by a Durbin-Watson statistic of 1.989. We found a statistically significant interaction (b = 0.0054, SE = 0.0018 p = 0.003, *r*^*2*^ increase = 0.0054, *SE* = 0.0018 *p* = 0.003, *r*^*2*^ increase = 0.018). Simple main effects indicated that in the case of anxiety at the mean level, the dose effect was significant (*b* = 0.024, *SE* = 0.012, *p* = 0.04, 95% CI: 0.0009, 0.048). High anxiety resulted in a stronger dosage effect (*b* = 0.053, *SE* = 0.013, *p* = 0.0001, 95% CI: 0.028, 0.079). For low anxiety, the relationship was insignificant (*b* = -0.005, *SE* = 0.017, *p* = 0.78, 95% CI: −0.039, 0.029). This interaction is shown in Fig. 1, panel B. The Johnson-Neyman technique showed that an anxiety score of 6 and greater (calculated threshold: 5.30) resulted in a significant dosage effect. One hundred and seventy-eight participants scored above that threshold.

#### The relationship between autodiagnosis and GDT results

Having answers to the single question whether gaming significantly impedes the participants' functioning, we decided to check whether such a simplified diagnosis would match with the GDT results. The comparison of participants who answered this question affirmatively (n = 28; MGDT = 12.21 ± 4.03) with those who denied this statement (n = 433; MGDT = 7.11 ± 2.70) using the Welch test (due to unequal variance) gave statistically significant results (95% CI, 3.522 to 6.685), *t*(28.59) = 6.604, *p* < 0.001, *d* = 1.83. None of the people who met the criteria of GD responded negatively to the self-diagnostic question, while 25 of those who were not considered problematic gamers according to the GDT criteria self-identified their gaming as significantly hindering their lives.

## Discussion

The present study attempted to test the hypothesis that some mental health conditions, more specifically depression and anxiety, can play the role of not only a mediator, but also a moderator of the relationship between the dose of gaming and the risk of GD. This prediction was directly inspired by previous research^4,11^ and the interactional-transformational model^20^, and was theoretically grounded in the I-PACE model of behavioral addictive disorders, which includes the interaction of personality traits, global and behavior-specific individual characteristics and psychopathological features (eg, anxiety and depression). The model postulates two stages in which psychopathological features can play different roles, but our intention was to explore the early stage in which anxiety and depression are expected to play a role in antecedent factors. Therefore, we collected a sample of active gamers without intentionally looking for people suffering from gaming disorders. Specifically, we pose two interactional hypotheses that both depression and anxiety would independently act as moderators of the relationship between weekly gaming time and the risk of GD. In addition, we tested a dosage effect and a model that took into account both depression and anxiety and their interactions with gaming time. Our results confirmed the existence and low effect size of the dosage effect. This result is in line with the existing literature, except that for our data, the percentage of explained variance (3.3%) was exceptionally low but still statistically significant. Subsequently, we decided to also add anxiety and depression to the model. This increased the fit of the model (12% of explained variance), while indicating a statistically insignificant role for anxiety in the prediction of the risk of GD in such a model. Next, we analyzed a model in which we allowed interactions between depression and anxiety with gaming time. The results were partly surprising, as this model further improved the model fit and confirmed our prediction that mental health variables act as moderators of the dosage effect. However, this was only the case for anxiety, not depression. So far, a similar relationship has only been demonstrated twice, in the studies of Yu et al.^4^ and Koncz et al.^11^. However, in the case of Koncz’s research, a statistically significant interaction was found between the severity of depression and the gaming time, which our model did not confirm. The role of general anxiety as a moderator has never been tested before, but a specific form of anxiety (i.e., social anxiety) was shown to moderate this relationship^4,33^.

Finally, we tested our a priori hypotheses. We decided to take this step despite the fact that the previously presented model seemed to exclude the role of depression as a moderator of the dosage effect for two reasons. First, we verify our hypotheses, which we set primarily for explanatory purposes (understanding the potential moderating role of both variables) and not for strictly predictive purposes. The result obtained in the former step could be satisfactory if we were looking for a way to optimize the predictive capabilities of the GD risk identification model. It shows that when the interaction between anxiety and gaming time is included in the model, taking into account the data on the interaction of depression with gaming time does not significantly improve the predictive value. However, our goal was different: we wanted to explain whether these variables could act as moderators. Second, the results we obtained in the working model seemed to contradict the results of Koncz et al.^11^. However, they did not provide a conclusive answer. As Koncz did not collect information on anxiety, the hypothetical possibility that if such data were included in their model, the results would be similar to those obtained by us cannot be ruled out. Therefore, testing the model with depression alone seemed like a logical step to bring us closer to clarifying this apparent inconsistency.

The results obtained confirmed the hypotheses. Both anxiety and depression, treated separately, played the role of moderators of the dosage effect. First, these results confirm and extend the finding of Koncz's team, which previously demonstrated the moderating role of depression in a sample of children^11^. In our case, a similar result occurred in a sample of adult gamers. Second, they also broaden previous findings on social anxiety as a moderator^4,33^; In our case, we were able to demonstrate a similar role for general anxiety. Third, such results fit the predictions of the I-PACE model, which includes psychopathological factors, such as mood disorders, among the factors that determine the risk of developing GD at an early stage. As can be seen, treating psychopathological factors as moderators can contribute to a better understanding of the mechanisms of development of GD (and probably other behavioral addictions), which in turn should contribute to its more effective prevention and treatment. At the same time, it should be emphasized that the results we obtained refer only to a fragment of the comprehensive I-PACE model. Therefore, our study should not be treated as an attempt to verify the entire model. Future research should attempt to test the postulates of the entire I-PACE model, unlike our study, which isolated only some of the variables. An additional effect of our study was the opportunity to explore to what extent the results obtained using GDT correspond to a simple self-diagnosis based on one question. We asked participants to indicate whether they agree with the statement ‘I think I play games in a way that significantly impairs my functioning’ with the answer options ‘yes’ and ‘no’, which can be treated as the most simplified way to identify people at risk of GD. Comparison of the average GD score between groups who directly stated that gaming had a negative impact on their functioning confirms these expectations: such people obtained average GDT scores almost twice as high as those who denied it, and this was also confirmed by a very high effect size. None of the respondents who met the recommended risk criteria according to the GDT (answer "4" or "5" to each of the four questions^26^) answered negatively to a single self-diagnostic question. However, what may be interesting is the relatively large number of people who self-identify as having a gaming problem who were not classified as such based on their GDT score. There were 25 such people, that is, almost 5% of the sample, which is in line with the previous study^26^. This result certainly confirms that it is necessary to have validated and nuanced screening tools because the general opinions of the respondents may not reflect reality. On the other hand, in the future it would be necessary to identify the reason why the assessment of the impact of gamers who feel that their gaming is harmful is not always reflected in the GDT result.

## Limitations

The present findings are subject to some limitations. The present study used data from a snowball procedure, which could be improved using a representative population sample. When recruiting respondents, we did not inform them in any way that the purpose of the study was related to GD, we did not establish any conditions regarding the intensity of playing; the only prerequisite was that the candidate played video games. This may also be related to the small number of respondents who met the recommended criteria to be considered at risk of GD; In a comparable study^26^, the percentage was three times higher, although it should be noted that in the case of a sample of 500 respondents, the differences between the result of 0.5% and 1.8% come down to the difference of several people in absolute terms. However, this means that our results must be treated with caution. Furthermore, it should be noted that our research was carried out using self-reports. This may be particularly important for gaming time estimates, which were retrospectively assessed. For this reason, the results we obtain may differ from the actual gaming time. In the future, the focus should be on eliminating this risk by circumventing participants' uncertain testimonies; perhaps it would be a good idea to collect objective data from them, for example, by accessing their user profiles on gaming websites or by recording activity on their devices. Unlike other researchers, we did not collect data on gaming time divided into weekdays and weekends; such a division is now standard and should also be introduced in studies continuing our direction^34^. The cross-sectional design allows us to only explore associations and prevents any attribution of causality. We decided not to collect data from minors, which should certainly be included in possible replication. This may be of particular importance, as the symptom profiles of mental disorders can differ between adults and adolescents^35^.

## Conclusions

In line with our hypotheses and the results of our predecessors, not only anxiety, but also depression, turned out to play the role of dosage effect moderators. Our findings were found to be consistent with the I-PACE model^21^. This result may prove to be very important in practice, as it seems to place the studied mental health conditions at the right place, not direct causes of the development of GD, but rather as genuine risk factors that can only contribute to GD when combined with a crucial trigger, gaming.

## Data Availability

The data sets used and analyzed in this study are available from the corresponding author upon reasonable request.
